# Activity-Limiting Musculoskeletal Conditions in US Veterans Compared to Non-Veterans: Results from the 2013 National Health Interview Survey

**DOI:** 10.1371/journal.pone.0167143

**Published:** 2016-12-22

**Authors:** Ramon Hinojosa, Melanie Sberna Hinojosa

**Affiliations:** Department of Sociology, University of Central Florida, 4297 Andromeda Loop North, Howard Phillips Hall 406, Orlando, FL, United States of America; University of Florida, UNITED STATES

## Abstract

Past military service is associated with health outcomes, both positive and negative. In this study we use the 2013 National Health Interview Survey to examine the constellation of conditions referred to as musculoskeletal disorders (MSDs) for Veterans and non-veterans with health conditions that limit their daily activities. Multivariate logistic regression analysis reveal that Veterans are more likely to report MSDs like neck and back problems, fracture bone and joint problems as an activity limiting problem compared to non-veterans. The relationship between age and reports of activity limiting MSDs is moderated by Veteran status. Veterans in this sample report more activity limiting MSDs at younger ages compared to non-veterans and fewer MSDs at older ages. This research contributes to our understanding of potentially limiting health conditions at earlier ages for Veterans.

## Introduction

There are 22 million Veterans of the United States Armed Forces [1], making Veterans an important subpopulation for health research [2]. Past military service is associated with health outcomes, both positive and negative. One area of concern for Veterans is musculoskeletal health. Research indicates that, compared to non-military civilians, Veterans are more likely to have musculoskeletal disorders such as arthritis, lower-back and hip and knee pain that impair mobility [3]. Musculoskeletal disorders (MSDs) are the most common cause of chronic pain and long-term physical disability in human populations [4]. Research from Canada, the United States, and Western Europe indicates the prevalence of physical disabilities caused by musculoskeletal disorders to be an estimated 4–5% of the total adult population [5]. According to the World Health Organization (WHO) musculoskeletal conditions represent some of the most physically disabling health conditions [4]. The WHO identified four musculoskeletal conditions as major disabling health conditions: osteoarthritis, rheumatoid arthritis, osteoporosis, and back pain. The most commonly reported MSDs worldwide are back pain (29%), osteoarthritis and osteoporosis (17%), rheumatoid arthritis (8%), ankle/foot pain (8%), knee pain (6%), hip pain (5%), shoulder pain (5%), hand/wrist pain (3%), and elbow pain (3%) [4]. Given that the 22 million U.S. Veterans represent approximately 7% of the total American population [1], understanding differences in MSDs between Veterans and non-military civilians and the ages at which these differences emerge is important and timely. We examine the constellation of conditions referred to as musculoskeletal disorders (MSDs) for Veterans and non-veterans using the 2013 National Health Interview Survey.

### Healthy Soldier, Healthy Warrior, Lower Mortality

Military service has both positive and negative health effects. Early in life military service members tend to be healthier than same-aged non-military counterparts [6]. This is partly due to the self-selection of the healthiest and fittest members of the civilian population into military service and partly the result of the rigorous physical demands associated with military training and service [7]. Thus, active duty service members tend to be more physically fit and less likely to be overweight compared to same-aged non-military civilians [6]. This phenomenon has been referred to as the “healthy soldier effect” (HSE) [6]. The healthy soldier effect is related to the “healthy warrior effect” (HWE), or the idea that the healthiest military personnel are the ones who are deployed [6]. Taken together the HSE and HWE highlight the ways military service might be protective of overall health for individuals after they leave military service. The literature on HSE and HWE support this by using standardized risk mortality ratios to indicate that Veterans’ risk of disease mortality is lower than non-military civilians on a range of health conditions [6, 8–10]. The protective effects of military service on disease mortality appear to last from 10 to 30 years after military service has ended [11].

### Musculoskeletal Problems and the Veteran

Standardized risk mortality ratios as the marker of health outcomes suggests that military service is health positive for most Veterans. If, however, health is viewed from the standpoint of morbidity, Veterans are more likely than similar aged civilians to have worse health. Studies indicate that Veterans have more cardiovascular disease [12], obesity [13, 14], and musculoskeletal disorders that limit mobility [3] than non-veterans. Studies of Canadian Veterans show disability related to MSDs that is 2.6 times higher than non-veterans [15] and rates of daily chronic pain that range from 25% to 72% of the Veteran population [16, 17]. For Veterans, who make-up approximately 25% of the American population over the age of 65 [18], concerns over the nature and type of chronic health condition and the age at which these conditions first appear is an important line of inquiry.

### Measuring Health in the Veteran Population

Most studies of the health of U.S. service members typically include data on active-duty service members or data on Veterans enrolled in the Department of Veteran Affairs (VA) health care system [13, 19]. There are three major critiques applied to studies of Veterans that use only data from the VA healthcare systems. First, this data captures Veterans who are in poorer health [20] and are more likely to have a disability rating than non-enrolled Veterans [21]. A disability rating is a rough measure of the level of physically incapacitating disability due to service-connected injury, illness, or disease [22]. Of those VA-enrolled Veterans with a disability rating, roughly 60% had a rating higher than 30% [21] indicating a significant level of physical disability. Second, VA enrolled Veterans are older than non-VA enrolled Veterans. Veterans over the age of 75 are more likely to enroll in and use VA services than younger Veterans and more likely to have chronic health conditions in need of ongoing care [23]. Third, most Veterans are not enrolled in VA health care services [24]. As of 2012, only 40% of eligible Veterans accessed and used VA services [23]. This leaves the possibility that use of VA-only data might provide a skewed picture of Veteran morbidity.

Large national studies comparing Veterans and non-veterans have until very recently been based on data collected before 2004 [2] missing many of the 2.5 million Americans deployed to Afghanistan and Iraq [1, 25]. Research indicates that these newest Veterans may have a unique disease trajectory that differs markedly from Veterans of other wars [26]. And finally, previous research tends to average health effects across age ranges by comparing Veterans, as a group, to non-veterans, as a group. This gives us little to no insight on when problems emerge or how they might vary by age.

The current study seeks to fill an important gap in the literature by using the National Health Interview Survey, a nationally representative data set that includes a representative sample of Veterans living in the civilian population to explore four MSDs leading to limitations of activity [27]; Back/Neck Problems; Fracture, Bone/Joint Injury; Other Musculoskeletal; Arthritis/Rheumatism. We compare Veterans and non-veterans by age and controlling for important sociodemographic characteristics.

## Materials and Methods

### Data

We used the 2013 National Health Interview Survey (NHIS) to examine the health of military Veterans in comparison to non-veterans with regard to MSDs. The NHIS is one of the nation’s largest cross-sectional household health surveys and is conducted through the Centers for Disease Control and Prevention’s National Center for Health Statistics (NCHS) [28]. The NHIS is a nationally representative sample of non-institutionalized individuals living in the United States. Excluded populations are active duty military personnel (who are not “Veterans” because they are on active-duty), patients in long-term care facilities, incarcerated individuals and foreign nationals.

### Sample

The NHIS follows a stratified multi-stage cross sectional design that is designed to produce estimates for the nation, for each of the four census regions and within census region by metropolitan status[28]. In the 2013 survey year, a total of 104,520 persons were surveyed within 42,321 families. The total household response rate for the entire survey was 75.7%. Of the 24.3% of non-responders, 16.3% of householders refused to be interviewed or the interviews were partial or unacceptable in some way. The remaining 8.0% were due to the failure of interviewers to locate the eligible respondents at their home after multiple attempts[27]. Individuals were surveyed in their home by a trained worker contracted through the Centers for Disease Control and Prevention (CDC). We were interested in the smaller subset (n = 11,532) of individuals who reported having a health condition which limits daily activities. A secondary question (“What causes your limitation?”) allowed respondents to mention any number of health conditions. From the list of health conditions identified by respondents, we identified 4 musculoskeletal disorders from the coded list to serve as our outcome variables for the analysis.

### Variables

#### Veteran Status

Respondents were asked whether they have ever served in the United States Armed Forces, Reserves, or National Guard. Those who identified as having ever served are called Veterans for the purposes of this study. This differs somewhat from Veteran status defined according to American legal code (38 U.S. Code § 101) as military personnel that have “served in the active military, naval, or air service” and were “discharged or released therefrom under conditions other than dishonorable”, which establishes the legal definition to determine eligibility for Veterans’ Benefits [29].

#### Health Conditions

The health conditions selected for analysis are back and neck problem; fracture, bone and joint injury; other musculoskeletal disorders; and arthritis. Table 1 below details the *International Classification of Disease 9*^*th*^ *edition* (ICD-9) codes used by the NHIS to create each musculoskeletal category (See Table 1).

**Table 1:** Musculoskeletal Conditions Defined by National Health Interview Survey 2013.

Condition	Description	ICD-9-CM Codes
**Back/Neck Problem**	Intervertebral disc disorders, other disorders of cervical region, other and unspecified disorders of back, osteochondropathies, curvature of spine	722 to 724.9, 732.0, 737 to 737.9
**Fracture, Bone/Joint Injury**	Fracture of skull, spine, trunk, upper/lower limb, dislocation, sprains/strains of joints and adjacent muscles OR other closed or open wound, poisoning with mention of bone/joint injury	800 to 848.9 or 850 to 999.9
**Other Musculoskeletal**	Other musculoskeletal system and connective tissue conditions, arthropathies and related disorders, dirsopathies, rheumatism, excluding the back, osteopathies, chondropathies, and acquired musculoskeletal deformities, fibromyalgia, lupus, osteoporosis, tendonitis and knee problems (excluding arthritis and joint injury)	710 to 739.9
**Arthritis/Rheumatism**	Arthropathy, crystal arthropathies, rheumatoid arthritis and other inflammatory polyarthropathies, osteoarthrosis and allied disorders, other and unspecified arthropathies, ankylosing spondylosis and other inflammatory spondylopathies, spondylosis and allied disorders, other disorders of soft tissues	711 to 712.9, 714 to 716.9, 720.0, 721 to 721.9, and 729.0

#### Sociodemographic Characteristics

Race and ethnicity was coded using the NHIS race/ethnicity recoded variable which merges respondents race (White, Black, Other) with their ethnicity (Hispanic, Non-Hispanic). This recoding divides the sample into racial and ethnic categories that include White, non-Hispanic; Black/African American, non-Hispanic; and Hispanic/Latino. We chose to exclude individuals who identified themselves as Other race, American Indian, Pacific Islander, Asian American because there were so few respondents in the analytic sample who self-identified using these categories. For example, there were 16 veterans who identified as Asian American and 24 respondents who fell into all other racial/ethnic categories (representing only 1% and 1.5% of the Veteran sub-sample, respectively). Sex was coded so that 1 = males, and 0 = females. Marital status was coded so that people who were married and those living with an unmarried partner (i.e., cohabiting) were coded in the same category, divorced and separated respondents were coded together, and widowed and never married respondents were each in their own category. Individuals with chronic health conditions that limit their daily activities are less likely to work for pay compared to those without chronic illnesses[30]. Therefore, we selected educational level as a proxy measure of socioeconomic status for this analysis. Educational level was coded so that 1 = less than a high school (HS) diploma, 2 = HS diploma, or general equivalency degree (GED), 3 = some college or 2 year college degree, 4 = 4 year college degree, 5 = graduate work or graduate degree. Age is treated as a continuous variable that ranges from 18 to 85+. In the NHIS data, age is top coded so that the public release data does not identify individuals above the age of 85 years.

#### Interaction terms

In order to test the moderating effect of race/ethnicity and age on the relationship between Veteran status and MSDs, we created 2 interaction terms in the analysis. The first is product term of Veteran status and race/ethnicity (white, black, Hispanic) and the second is a product term for Veteran status and age (in years).

### Analytic Strategy

**Fig 1:**
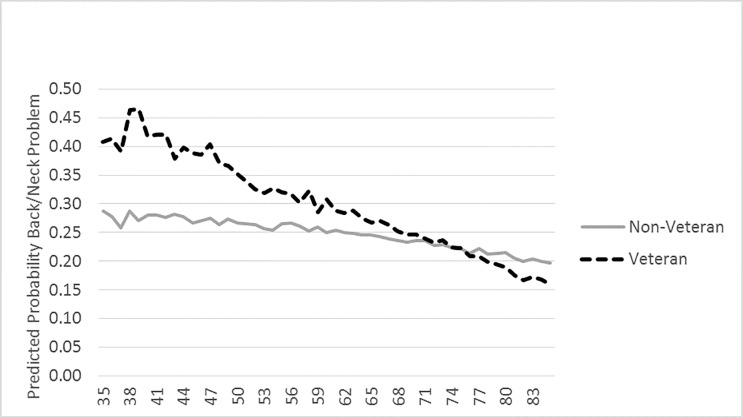
Graphic Representation of the Interaction Between Veteran Status and Age on Back/Neck Problem.

**Fig 2:**
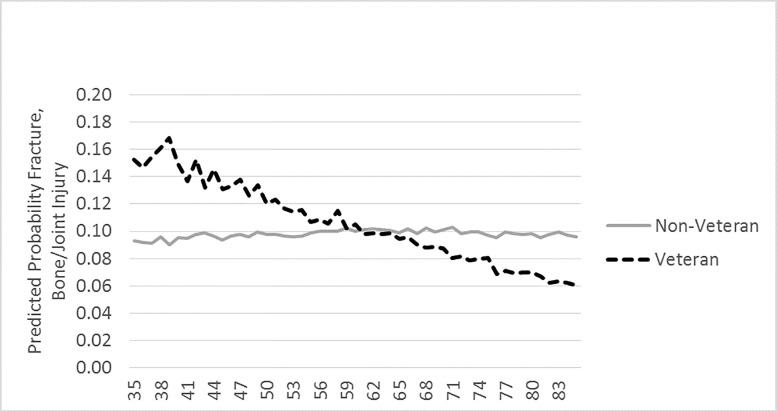
Graphic Representation of the Interaction Between Veteran Status and Age on Fracture, Bone/Joint Injury.

All analyses were run using SPSS version 23.0 [31]. In this analysis, we ran bivariate tests (Chi-square and T test) to examine the difference between Veterans and non-veterans across sociodemographic characteristics. Table 2 displays the results of the bivariate tests. Next, we ran a series of logistic regression models to obtain the odds of reporting one of four MSDs controlling for sociodemographic characteristics. The numbers presented in the columns of Tables 3 and 4 are the odds ratios and the 95% confidence interval around the odds ratios. The first bivariate logistic regression models examined the likelihood of reporting a health condition by Veteran status. The second multivariate logistic regression models included educational level, income, marital status, race/ethnicity, gender, and age. Fig 1 and Fig 2 in the results section depict the predicted probability of reporting MSDs in the fully adjusted logistic regression models by age and Veteran status. All models are weighted taking into account the NHIS survey recommendations for complex sample designs. In SPSS version 23, a complex samples plan file was created that accounted for the variance estimation of the stratum (STRAT_P) and primary sampling unit (PSU_P) as well as the final person file weight (WTFA). All logistic regression analyses were run using the complex samples CSLOGISTIC command.

**Table 2:** Unweighted and Weighted Distributions of Sociodemographic Variables by Veteran Status.

	Non-Veterans				Veterans				
	Unweighted N = 9,084				Unweighted N = 1535				
	Weighted N = 27,771,046				Weighted N = 4,808,827				
	Unweighted N	Unweighted %	Weighted N	Weighted %	Unweighted N	Unweighted %	Weighted N	Weighted %	
Marital Status*									
Married/Living with Partner	3851	42.4%	12027394	43.09%	894	58.2%	2761203	57.4%	*
Widowed	1662	18.3%	5165824	18.6%	231	15.0%	780994	16.2%	
Divorced/Separated	1838	20.2%	5413510	19.5%	307	20.0%	959399	20.0%	
Never Married	1733	19.1%	5164318	18.6%	103	6.7%	307231	6.4%	
	**9084**	**100.0%**	**27771046**	**100.0%**	**1535**	**100.0%**	**4808827**	**100.0%**	
Educational Level*									
Less than HS	2509	27.6%	6889681	24.8%	207	13.5%	607874	12.6%	*
HS graduate or GED	2991	32.9%	9422001	33.9%	469	30.6%	1456863	30.3%	
Some College	2372	26.1%	7444133	26.8%	564	36.7%	1761793	36.6%	
4 Year College Degree	831	9.1%	2751439	9.9%	183	11.9%	602551	12.5%	
Graduate Work	381	4.2%	1263792	4.6%	112	7.3%	379746	7.9%	
	**9084**	**100.0%**	**27771046**	**100.0%**	**1535**	**100.0%**	**4808827**	**100.0%**	
Race/Ethnicity*									
White	5946	65.5%	20587448	74.1%	1218	79.3%	4050137	84.2%	*
Black/African American	1784	19.6%	2992486	10.8%	233	15.2%	556214	11.6%	
Hispanic/Latino	1354	14.9%	4191112	15.1%	84	5.5%	202476	4.2%	
	**9084**	**100.0%**	**27771046**	**100.0%**	**1535**	**100.0%**	**4808827**	**100.0%**	
Sex of Respondent*									
Male	3172	34.9%	9770135	35.2%	1439	93.7%	4519433	94.0%	*
Female	5912	65.1%	18000911	64.8%	96	6.3%	289394	6.0%	
	**9084**	**100.0%**	**27771046**	**100.0%**	**1535**	**100.0%**	**4808827**	**100.0%**	
	Unweighted		Weighted		Unweighted		Weighted		
Age in Years*	57.8		58.1		66.7		67.34		*

* Indicates a significant difference between Veterans and Non-Veterans in Chi Square or T Test at p < .05

**Table 3:** Logistic Regression Models For Odds of Reporting an Activity Limiting Musculoskeletal Disorder.

	Back/Neck Problem						Arthritis/Rheumatism					
	Unadjusted			Adjusted			Unadjusted			Adjusted		
	OR	Upper	Lower	OR	Upper	Lower	OR	Upper	Lower	OR	Upper	Lower
Veteran	1.07	0.93	1.24	2.80	1.38	5.70	0.98	0.85	1.13	1.95	0.86	4.38
Non-Veteran (Ref.)												
Black				0.80	0.68	0.93				1.23	1.05	1.43
Hispanic				0.86	0.73	1.01				0.96	0.81	1.14
White (Ref.)												
Female				0.91	0.81	1.02				1.69	1.49	1.93
Male (Ref.)												
Married/Living with Partner				2.05	1.68	2.50				1.26	1.03	1.53
Widowed				1.76	1.36	2.28				1.37	1.08	1.73
Divorced/Separated				2.16	1.75	2.68				1.60	1.30	1.97
Never Married (Ref.)												
Age of Respondent				0.99	0.99	0.99				1.03	1.03	1.04
Less than HS Graduate				1.40	1.05	1.87				1.27	1.00	1.62
HS Graduate				1.33	1.01	1.75				1.00	0.78	1.28
Some College				1.64	1.25	2.15				1.09	0.85	1.39
4 Year College Degree				1.28	0.94	1.75				0.87	0.65	1.16
Graduate/Professional Degree (Ref.)												
Veteran * Black				0.99	0.99	0.99				1.17	0.79	1.74
Veteran * Hispanic				1.16	0.64	2.08				1.24	0.68	2.25
Veteran * White (Ref.)												
Veteran * Age of Respondent				0.98	0.97	0.99				0.99	0.98	1.00
Intercept	0.32	0.30	0.34	0.27	0.19	0.38	0.31	0.29	0.33	0.02	0.01	0.03
Nagelkerke Pseudo R Squared	0.00			0.03			0.00			0.11		

Ref. = Reference group; OR = odds ratio; Upper/Lower = upper and lower limits of the 95% confidence interval for odds ratio

**Table 4:** Logistic Regression Models For Odds of Reporting an Activity Limiting Musculoskeletal Disorder.

	Back/Neck Problem						Arthritis/Rheumatism					
	Unadjusted			Adjusted			Unadjusted			Adjusted		
	OR	Upper	Lower	OR	Upper	Lower	OR	Upper	Lower	OR	Upper	Lower
Veteran	0.95	0.78	1.15	**3.45**	**1.41**	**8.40**	**0.81**	**0.68**	**0.98**	1.37	0.53	3.55
Non-Veteran (Ref.)												
Black				**0.74**	**0.58**	**0.96**				0.99	0.80	1.21
Hispanic				1.01	0.79	1.30				**1.24**	**1.01**	**1.53**
White (Ref.)												
Female				**0.83**	**0.72**	**0.95**				**1.84**	**1.56**	**2.17**
Male (Ref.)												
Married/Living with Partner				1.24	0.98	1.58				**1.57**	**1.27**	**1.95**
Widowed				1.20	0.84	1.72				1.27	0.95	1.70
Divorced/Separated				**1.46**	**1.11**	**1.91**				**1.62**	**1.27**	**2.06**
Never Married (Ref.)												
Age of Respondent				1.00	1.00	1.01				1.00	1.00	1.01
Less than HS Graduate				1.07	0.72	1.59				1.01	0.75	1.38
HS Graduate				1.09	0.75	1.59				0.94	0.68	1.31
Some College				1.29	0.87	1.90				1.11	0.81	1.53
4 Year College Degree				1.40	0.92	2.12				0.99	0.71	1.38
Graduate/Professional Degree (Ref.)												
Veteran * Black				1.16	0.65	2.05				1.04	0.62	1.76
Veteran * Hispanic				1.23	0.53	2.84				0.94	0.47	1.91
Veteran * White (Ref.)												
Veteran * Age of Respondent				**0.98**	**0.96**	**0.99**				1.00	0.98	1.01
Intercept	**0.11**	**0.10**	**0.12**	**0.08**	**0.05**	**0.13**	**0.16**	**0.15**	**0.17**	**0.06**	**0.04**	**0.09**
Nagelkerke Pseudo R Squared	0.00			0.01			0.00			0.03		

Ref. = Reference group; OR = odds ratio; Upper/Lower = upper and lower limits of the 95% confidence interval for odds ratio

## Results and Discussion

Table 2 displays both the raw and weighted characteristics of our sample by Veteran status. We report on the weighted characteristics of the sample. Veterans represented approximately 17% of the total sample of individuals reporting activity limitations. Veterans in the sample are older, with a mean age of 67.3 years compared to 57.8 for non-veterans. More Veterans are married or living with a partner than non-veterans, and Veterans had higher levels of education with 57% completing some college or above compared to only 41.3% of non-veterans. Veterans are more likely to be white and male compared to non-veterans.

Table 3 displays the results of the multivariate logistic regression. The results of the unadjusted models (with only Veterans status) are shown alongside the adjusted models (adjusting for sociodemographic characteristics including educational level, income, marital status, race/ethnicity, gender, and age) for Back/Neck Problem andArthritis/Rheumatism. Table 4 displays the results for Fracture, Bone/Joint Injury, or Other Musculoskeletal Condition along with the odds ratios and 95% confidence intervals around the odds ratio for each of the models.

### Back/Neck Problem

In the adjusted models (Table 3), Veterans are almost 3 times more likely to report a back/neck problem compared to non-veterans (OR = 2.80, 95% CI 1.38 to 5.70). Age was found to be a factor in back/neck problem in the sample, with a negative relationship between age and the odds of reporting back/neck problems (OR = .99, 95% CI .99 to .99). For every one unit increase in Veterans age, the odds of reporting a back/neck problem go down by 1% compared to same-aged non-veterans. Respondents who were married/living with a partner, widowed or divorced/separated all had a significantly higher likelihood of having back/neck problems compared to respondents who were had never been married. Respondents with less than a high school education, high school graduates, and respondents with some college had a higher odds of having back/neck problems compared to respondents with a graduate or professional degree. Black respondents had a lower odds of reporting back/neck problem compared to whites (OR = .80, 95% CI .68 to .93). We also tested for a moderating effect of race/ethnicity on the relationship between Veteran status and back/neck problems. Black Veterans have a lower odds of reporting back/neck problems that is 1% lower compared to white Veterans (OR = .99, 95% CI .99 to .99).

### Arthritis/Rheumatism

In the model that includes only veteran status (Table 3), we see that Veterans are as likely as non-veterans to report arthritis/rheumatism as an activity limiting health condition. The same was true of the models adjusted for sociodemographic characteristics; Veterans were just as likely as non-veterans to report arthritis/rheumatism. Black respondents were more likely than white respondents to report arthritis/rheumatism (OR = 1.23, 95% CI 1.05 to 1.43) as were female compared to male respondents (OR = 1.69, 95% CI 1.49 to 1.93). For every one year increase in age, there is a corresponding 3% increase in the odds of reporting arthritis/rheumatism for both Veterans and non-veterans (OR = 1.03, 95% CI 1.03 to 1.04). Never married respondents were less likely to report arthritis/rheumatism compared to those who are married/living with a partner, widowed, or divorced/separated. Educational level was not a significant predictor of arthritis/rheumatism, nor was the interaction between Veteran status and race/ethnicity.

### Fracture, Bone/Joint Injury

In the unadjusted models (Table 4), the results indicate that Veterans are just as likely as non-veterans to report a fracture, bone or joint injury as an activity-limiting health condition. In the models adjusted for sociodemographic characteristics, Veterans were nearly 3.5 times more likely to report an activity-limiting fracture, bone/joint injury compared to non-veterans (OR = 3.445, 95% CI 1.41 to 8.39). Black respondents were less likely to report a fracture, bone, or joint injury compared to whites (OR = 0.74, 95% CI 0.58 to 0.96) and females were less likely compared to males (OR = .83, 95% CI 0.72 to 0.95). Divorced and separated respondents had a higher odds of reporting a fracture, bone, or joint injury compared to never married respondents (OR = 1.46, 95% CI 1.11 to 1.91).

### Other Musculoskeletal

In the unadjusted models, results indicate that Veterans have a lower odds of reporting other (OR = 0.81, 95% CI 0.68 to 0.98), being 19% less likely to report other musculoskeletal conditions compared to non-veterans (Table 4). In the adjusted models, however, Veterans and non-veterans are equally likely to report other MSDs. There were differences by race/ethnicity, with Hispanic respondents more likely than non-Hispanic whites (OR = 1.24, 95% CI 1.01 to 1.53) to report other MSDs. And in general, female respondents are more likely than male respondents to report other MSDs as activity-limiting (OR = 1.84, 95% CI 1.56 to 2.17). Married and divorced/separated respondents are more likely to report other MSDs compared to never married respondents. There is no difference in the odds of reporting other MSDs by Veteran status and age.

### Veteran Status and Age

Veteran status is a significant moderator for the relationship between age and two MSDs—back/neck problems and fracture, bone/joint injury. In order to understand the moderating effect, we have plotted the mean predicted probability of having these MSDs for each age by Veteran status, Figs 1 and 2 display this relationship.

Among both Veterans and non-veterans, as shown in Fig 1, the predicted probability of reporting back/neck problems decreases with age, but Veterans start out with a higher predicted probability. The largest gap appears at ages 38 and 39, with almost double the chance of reporting an activity limiting back and neck problem. At ages 71 and 72 Veterans and non-veterans converge, after which age Veterans have a lower predicted probability compared to non-veterans. Fig 2 shows that the pattern of predicted probabilities for fracture, bone/joint injury is shaped like an “X” with Veterans having higher odds of reporting fracture, bone/joint injury at younger ages, but lower odds at higher ages. The figure also shows that among non-veterans, the odds of reporting fracture, bone/joint injury as they age is relatively flat. For Veterans, there are decreasing odds with increasing age. The largest difference appears at age 39 when Veterans predicted probability of reporting such an injury is .17; for non-veterans it is .10. Veteran and non-Veteran predicted probability converge at ages 59 to 64, after which Veterans have a lower probability of reporting fracture, bone/joint injuries.

Military service has both positive and negative health effects for Veterans. Our findings show that Veterans are almost 3 times more likely to report a back/neck problem compared to non-veterans. As indicated above, there was a *decreasing odds* of Veterans reporting a back/neck problem with advancing age compared to same-aged civilians. This suggests that Veterans have back and neck problems at younger ages, but the aging process appears to erase the higher likelihood of reporting as non-veterans eventually catch up physically to Veterans. The source of the back and neck injury is not reported, but the main difference between Veterans and non-veterans is military service. This is unsurprising given that military service-related duties consist of regular, sustained physical activity and intensive repetitive physical labor, both common sources of musculoskeletal injuries. The risks for military-related injuries begin in basic training with regular physical training that continues throughout the Veteran’s military career. State-side military post duty carries its own risks resulting from the demands of the military occupational specialty (MOS). Logistics occupations require regular lifting and movement of supplies and equipment. Communications and monitoring specialties require long periods of sitting, which may put troops at risk for musculoskeletal problems resulting from poor posture. Medical service personnel may walk long hours on hard tile floors in hospitals and clinics in addition to pushing, pulling, rolling, and transporting heavy loads of supplies and patients. Pre-deployment activities offer unique musculoskeletal risks that stem from the intensive period of readying and centralizing war equipment. During deployment neck and back injuries can result from a number of sources; long 12–16 hour workdays that translate into more lifting, sitting, or pushing, than regular stateside post duty. Protective gear like the PASGT helmet, which weighs 3 to 4 lbs depending on size, puts strain on the soft tissues of the neck and back. Life on a forward operating base sometimes means unexpectedly needing to run, jump and sprint on uneven to avoid incoming fire. Troops assigned combat patrol may regularly do this while also carrying 40 to 120 lbs. or more of supplies and equipment. Deployment also places troops at increased risk of small arms fire or blast-related musculoskeletal injuries. Evidence suggests that injuries are more likely to be related to the more mundane experiences related to MOS duties rather than from direct combat [32, 33]. One study found that being in a Calvary or Infantry unit, wearing body armor more than 6 hours a day, spending more than 21 hours a week in a tactical vehicle, or walking patrol more than an hour a day predicted episodes of lower back pain in soldiers fighting in Afghanistan. Soldiers spending 1 to 4 hours a day at a desk and more than 5 hours a day lifting were also likely to have lower back pain [34].

Our analysis also found that respondents who were married/living with a partner, widowed or divorced/separated all had a significantly higher likelihood of having back/neck problems compared to respondents who were had never been married. This likely captures an age dynamic, as younger persons are *less likely* to be married, divorced, or widowed in general. Younger bodies are more resistant to musculoskeletal injuries and are not as likely as older bodies to suffer injuries resulting from chronic overuse that comes with the aging process. Along these lines, other research has shown that among active duty personnel suffering from lower back pain, older service members had higher rates of back pain compared to younger service members [35]. We have no way of knowing the age at which the neck/back injury occurred and cannot say whether it happened during or after military service. Given the Healthy Soldier Effect, or the tendency of the healthiest and most physical fit to enlist in the military, it is unlikely these musculoskeletal injuries occurred prior to military service. Whenever the back and neck injuries occurred, they were more likely to be reported by older service members, those who, generally speaking, are the most likely to be married, separated, or divorced.

We also explored the interaction between Veteran status and race/ethnicity. We found that Black Veterans specifically had lower odds of reporting back/neck problems compared to white Veterans and Hispanics. Black military personnel tend to be overrepresented in logistics support and administrative services compared to whites and Hispanics who tend to be overrepresented in combat specialties [36]. Hispanics were more likely to be deployed to a combat zone than non-Hispanic Whites and Blacks (42.9% for Hispanics, 33.5% for Whites, and 34.6% for Blacks) [37]. The different MOS related risks and lower likelihood of deployment for Blacks may help explain some of the differences in reporting among Veterans.

Veterans were more than 3 times more likely to report an activity-limiting fracture, or bone/joint injury compared to non-veterans in the adjusted models. Black respondents were also less likely to report fracture, or bone/joint injury as an activity limitation compared to whites. Like the reporting of back/neck injuries, military service predisposes Veterans to injury via training, deployment, and/or combat. Damage to bones and joints is one possible result.

There was no relationship in our study between Veteran status and reporting Arthritis/Rheumatism or Other Musculoskeletal Conditions as an activity limiting condition. This differs from other research that suggests Veterans report higher rates of arthritis than civilians [3, 38]. Murphy et al. (2014) used data from the Behavioral Risk Factor Surveillance System, another nationally representative data set that includes Veterans living in the general population, to report that Veterans had a higher overall prevalence of doctor-diagnosed arthritis than non-veterans, with an age-standardized rate of 25.6% for Veterans compared to 23.6% for civilians. For both men and women, arthritis prevalence was higher among veterans than non-veterans; for male Veterans, the overall rate of arthritis was 25%; for civilians, the rate was 19.5%. For female Veterans, the rate was 31.3% compared to 26.1% for civilians. Other studies have shown that arthritis is the most common chronic condition reported by Veterans [39]. Given that musculoskeletal injuries are a distinct risk-factor in the development of chronic arthritis, we anticipated a relationship between Veteran status and reporting Arthritis/Rheumatism as an activity limiting condition. The lack of significant findings in our study regarding Arthritis/Rheumatism can be explained by questioning whether arthritis is perceived by Veterans to be an activity limiting condition. Recall that we were interested in a smaller subset of respondents who reported having a limitation to their daily activities, and were then asked to state the health condition that was the source of their limitation. It may be that a diagnosis of Arthritis is present, but may not limit a Veterans’ daily function any more or less than non-veterans.

Our results indicate that Veterans with back and neck injuries and fracture, bone and joint injuries that limit activity suffer activity limitations at earlier ages than non-veterans. Musculoskeletal problems represent some of the most physically disabling chronic health conditions [4], and disability related to these conditions increases with age [40]. Although non-veterans eventually mirror Veterans’ rates of reported limitation, Veterans suffer longer years of musculoskeletal injury related limitations. These musculoskeletal problems will plague younger Veterans for much of their post-military lives with the potential to seriously impair health, well-being, and general overall quality of life for more years of their life.

### Limitations

The National Health Information Survey (NHIS) data do not allow for drilling down into the specifics of the musculoskeletal injuries. Data report the presence of a Back/Neck injury, but beyond this, we cannot determine whether the injury is to the neck or back, the upper back or lower back. We also cannot say whether musculoskeletal injuries are service-connected or occurred after military service was completed. Back/Neck and Fracture, Bone/Joint injuries are reported as activity limiting conditions by Veterans at younger ages and higher rates than non-veterans, but the specifics of where and when are inaccessible given the data.

As noted above, NHIS respondents who indicated they had ever served in the United States Armed Forces were coded as “Veterans”. The NHIS does not verify Veteran status using the legal definition, which according to 38 U.S. Code § 101 of U.S. law, is someone who has “served in the active military, naval, or air service” and was “discharged or released therefrom under conditions other than dishonorable”. The only available data that does confirm Veteran status comes from the Veterans Health Administration using VHA-enrolled Veterans; to be eligible for VA benefits, Veterans must meet the legal definition for Veteran status. One problem with VHA data is that the majority of eligible Veterans are not enrolled in VA health care services [24]. Another issue is that Veterans who are enrolled in VA care are in poorer health [20], more likely to have chronic health conditions, and are older than non-enrolled Veterans [23]. This skews out understanding of Veterans’ health issues. We also note that the legal definition for Veteran status is imperfect as several recent reports document wrongful discharge of Veterans under “Dishonorable” conditions, rending them ineligible for VA benefits [41–43]. The NHIS data provides a different picture of Veterans’ health partly because it is able to capture those Veterans who may not be represented in VHA data.

### Future Directions

This study established that Back/Neck and Fracture, Bone/Joint injuries are more likely to be reported as activity limiting conditions at younger ages for Veterans compared to non-veterans. Future research should explore the nature, type, and severity of reported activity limitations. For example, are activity limitations specific to activities of daily living, or ADLs—feeding, bathing, toileting, dressing, or are the reported limitations centered on instrumental activities of daily living (IADLs), such as the ability to do housework or participate in community functions? How many limitations in ADLs and IADLs are respondents experiencing? How do Veterans and non-veterans differ in their ADL and IADL limitations? Are there age-related differences in limitations of ADLs and IADLs? Such questions can more fully capture the ways that military service affects Veterans health.

We also highlighted significant differences for musculoskeletal injury-related limitations between Veterans and non-veterans by age. Future work will explore differences *among* Veterans by age, taking a more intersectional approach; for example sex-by-age-by-Veteran status. There is some evidence indicating that female OEF/OIF Veterans are more likely to be diagnosed with musculoskeletal problems than male OEF/OIF Veterans [44]. Expanding this work to all eras of female Veterans is a next logical step. Establishing a baseline for differences in male/female Veteran musculoskeletal health allows researchers to track over time any changes in musculoskeletal health, by sex, that may occur. This is timely given that, as of January 2016, combat-related MOSs are open to women, exposing future female Veterans to new risks of musculoskeletal injury related to expanded options for military training and duty.

In addition to sex-age-Veteran status interactions, looking at sex-by-race-by-Veteran status across age will provide important information on which categories of Veterans are most at risk for musculoskeletal injuries, they types of injuries they are most at-risk of suffering, and the ages at which they are most likely to report injury-related limitations.

## Conclusion

Veterans in this study are more likely at younger ages to report Back/Neck and Fracture, Bone, and Joint injuries as activity-limiting conditions compared to non-veterans. Using the National Health Information Survey provides a unique, nationally representative sample of Veterans that offers of broader picture of Veterans’ health concerns than is traditionally offered through the use of VA data alone. We offer this research as a general guide to thinking about the role that military service may play in the musculoskeletal health, and overall quality of life, of Veterans.

## Abbreviations

MSD: musculoskeletal disorders
NHIS: National Health Interview Survey
HSE: Healthy Soldier Effect
HWE: Healthy Warrior Effec
US: United States
VA: Veterans Affairs
OR: Odds Ratio
CI: Confidence Interval
ICD-9: International Classification of Diseases 9^th^ Edition
